# Integrative network pharmacology, transcriptomics, and molecular docking identify candidate *Centella asiatica* constituents and targets in neurodegenerative diseases

**DOI:** 10.1371/journal.pone.0354882

**Published:** 2026-07-31

**Authors:** Yuxi Xie, Chong-Teik Lim, Xin-Jieh Lam, Pike-See Cheah, King-Hwa Ling, Tan Huang

**Affiliations:** 1 Department of Clinical Nutrition, The Second Affiliated Hospital of Zunyi Medical University, Zunyi, Guizhou, China; 2 Department of Biomedical Sciences, Faculty of Medicine and Health Sciences, Universiti Putra Malaysia, Serdang, Selangor, Malaysia; 3 Department of Molecular Neuroscience, Graduate School of Medicine and Pharmaceutical Sciences, University of Toyama, Toyama, Japan; 4 Department of Human Anatomy, Faculty of Medicine and Health Sciences, Universiti Putra Malaysia, Serdang, Selangor, Malaysia; 5 Malaysian Research Institute on Ageing (MyAgeing®), Universiti Putra Malaysia, Serdang, Selangor, Malaysia; 6 M Kandiah Faculty of Medicine and Health Sciences, Universiti Tunku Abdul Rahman Cheras, Kajang, Selangor, Malaysia; 7 Fakultas Kedokteran, Universitas Pembangunan Nasional “Veteran” Jakarta, Jakarta, Indonesia; 8 Department of Neurosurgery, The Second Affiliated Hospital of Zunyi Medical University, Zunyi, Guizhou, China; Guangdong Nephrotic Drug Engineering Technology Research Center, Institute of Consun Co. for Chinese Medicine in Kidney Diseases, CHINA

## Abstract

**Background:**

Neurodegenerative diseases, including Alzheimer’s disease (AD), Parkinson’s disease (PD), and Huntington’s disease (HD), are progressive disorders with limited therapeutic options. *Centella asiatica* (*C. asiatica*), a medicinal and edible plant, has been reported to exert neuroprotective and anti-neuroinflammatory properties. Yet, the mechanisms underlying its effects against neurodegenerative diseases remain largely unclear.

**Methods:**

We employed an integrative strategy combining network pharmacology, transcriptomic analyses, machine learning and molecular docking to prioritize disease-associated molecular networks and candidate compound–target relationships in AD, PD and HD.

**Results:**

Sixteen candidate constituents of *C. asiatica* met the predefined drug-likeness, gastrointestinal absorption and blood–brain barrier permeability criteria, yielding 370 unique predicted targets. Disease-gene mining identified 983 AD-associated genes, 1,103 PD-associated genes, and 3,316 HD-associated genes. Integration of compound targets, disease-associated genes, and transcriptomic profiles prioritized five hub genes in PD (CCKAR, MAPK8, PSEN2, SLC6A3, and TH), four in AD (APP, PGK1, PIK3CA, and TTR), and four in HD (CHRND, HSP90AA1, PRKCQ, and TH). Enrichment analyses highlighted disease-relevant processes involving neurotransmitter signalling, cAMP and calcium pathways, MAPK-related responses and inflammatory regulation. ROC analyses provided additional support for the discriminatory performance of the prioritized genes in independent datasets, whereas molecular docking identified favourable predicted Vina docking scores and structurally plausible interactions between selected compounds and hub targets.

**Conclusion:**

This integrative computational analysis prioritizes candidate *C. asiatica* constituents, putative disease-associated targets, and molecular pathways in AD, PD, and HD. The findings provide a foundation for subsequent biochemical, cellular, and in vivo validation.

## Introduction

Neurodegenerative diseases impose a substantial global health burden, severely compromising the quality of life for millions of individuals worldwide, especially Alzheimer’s disease (AD) [1], Parkinson’s disease (PD) [2], and Huntington’s disease (HD) [3]. A unifying pathological hallmark across these disorders is the aberrant accumulation of misfolded proteins. In AD, extracellular amyloid-β plaques and intracellular neurofibrillary tangles disrupt synaptic architecture and neuronal function, ultimately contributing to progressive cognitive decline [4]. Similarly, PD is characterized by the aggregation of α-synuclein into Lewy bodies, which compromises neuronal integrity and precipitates the selective degeneration of dopaminergic neurons in the substantia nigra, resulting in both motor dysfunction and cognitive impairment [5]. In HD, the polyglutamine-expanded mutant huntingtin protein forms toxic intracellular inclusions that disrupt neuronal homeostasis, driving motor abnormalities, cognitive deficits, and psychiatric manifestations [6]. Notably, the aggregation mechanisms of these misfolded proteins share prion-like properties, whereby aberrant proteins can induce conformational changes in their normally folded counterparts, facilitating pathological propagation across neuronal circuits [7,8]. These abnormal protein assemblies are associated with a cascade of neurodegenerative processes, including chronic neuroinflammation, mitochondrial dysfunction, oxidative stress, synaptic loss, impaired autophagy, and disruption of protein homeostasis, ultimately culminating in widespread neuronal loss [4–6,9]. As such, early and effective intervention strategies are crucial for improving patient outcomes and mitigating the broader societal burden of these devastating disorders.

Current treatments for AD, PD, and HD are largely symptomatic and do not halt the underlying neurodegenerative process. Levodopa, the principal symptomatic treatment for PD, is associated with significant long-term adverse effects, most notably dyskinesia and other motor complications; the incidence of levodopa-induced dyskinesias may reach over 60% after more than five years of therapy [10]. The cholinesterase inhibitor Donepezil, used in AD, may cause gastrointestinal disturbances (nausea, vomiting, diarrhea), vagotonic cardiac effects (bradycardia, conduction block), and can predispose to syncope in high-risk patients [11]. These limitations have prompted continued interest in plant-derived compounds as potential sources of multi-target neuroprotective agents.

*Centella asiatica* (*C. asiatica*), a medicinal and edible herb belonging to the Apiaceae family, is widely distributed across Asian countries, such as China, Malaysia, and India. It has traditionally been employed to treat gastrointestinal disorders, dermatological conditions, infections, heatstroke, and cognitive and memory impairments [12–15]. Beyond its medicinal applications, *C. asiatica* is also integrated into dietary use: dried leaves are brewed into herbal teas, while fresh leaves are consumed as vegetables or juiced in various formulations [15,16]. More broadly, food–medicine homologous plants are increasingly investigated as sources of bioactive constituents with antioxidant, anti-aging and neuroprotective potential. Recent reviews of Phyllanthus emblica and food–medicine homologous leguminous species have highlighted oxidative-stress modulation and nervous-system protection as recurring biological themes within this class of medicinal foods [17,18]. Phytochemical investigations have revealed that extracts and candidate constituents of *C. asiatica* exhibit a wide spectrum of pharmacological activities, including anti-inflammatory, antioxidant, mitochondrial-protective, anti-apoptotic, and immunomodulatory effects [19–21]. Emerging studies have further demonstrated that several of its compounds can cross the blood–brain barrier (BBB) and exert neuroprotective effects [22,23]. These actions encompass modulation of neuroinflammatory responses, enhancement of neuronal energy metabolism, upregulation of neurotrophic factors, reinforcement of synaptic plasticity and excitatory neurotransmission, attenuation of neuronal aging and apoptosis, and improvement of cognitive performance [20,24–26]. While more than 100 chemical constituents have been identified in *C. asiatica* extracts, the current literature has primarily focused on only a limited subset, particularly asiaticoside, madecassoside, asiatic acid, and madecassic acid [27]. The broader neuropharmacological potential of the remaining compounds remains largely unexplored. Moreover, the precise molecular targets and signaling mechanisms underlying the potential biological actions of *C. asiatica* against neurodegenerative diseases are still insufficiently characterized.

In silico approaches have increasingly been applied to food–medicine homologous plants to explore their candidate bioactive constituents, putative molecular targets and potential biological relevance [28]. In this study, we integrated network pharmacology, transcriptomic analysis, machine-learning-based target prioritization and molecular docking to identify candidate *C. asiatica* constituents, putative hub targets and disease-relevant pathways in AD, PD and HD. This framework was designed to generate experimentally testable hypotheses regarding the potential neuroprotective actions of *C. asiatica*.

## Materials and methods

### Identification and screening of candidate constituents of *C. asiatica*

All chemical constituents of *C. asiatica* and their corresponding canonical SMILES strings were retrieved from the HERB database [29] (http://herb.ac.cn/). Pharmacokinetic and drug-likeness properties were evaluated using SwissADME [30] (http://www.swissadme.ch/). The screening set was defined as all *C. asiatica* constituents retrieved from HERB with available chemical structure information. Candidate compounds were then filtered using a predefined rule-based strategy based on Lipinski’s Rule of Five and predicted absorption/distribution properties. Compounds were retained only if they met all of the following criteria: molecular weight <500 Da, no more than five hydrogen-bond donors, no more than ten hydrogen-bond acceptors, logP between −2 and 5, no more than ten rotatable bonds, high predicted gastrointestinal absorption (GIA) and predicted blood–brain barrier (BBB) permeability. The final analysis set comprised compounds satisfying all predefined criteria. No compounds failing these criteria were manually included.

### Identification of *C. asiatica*–related targets

The SwissTargetPrediction database [31] (http://swisstargetprediction.ch/), which is specifically designed to predict the potential targets of bioactive small molecules, was employed to identify putative targets of the selected candidate constituents present in *C. asiatica*. The canonical SMILES strings of the selected compounds were uploaded to SwissTargetPrediction, with Homo sapiens specified as the target organism. The predicted target genes were then downloaded for further analysis. Subsequently, RStudio (Version: 2025.05.0 + 496) was used to clean and integrate the data, retaining only those targets with a prediction probability >0.05.

### Identification of disease-associated target genes

To identify disease-associated target genes, the keywords “Alzheimer’s disease”, “Parkinson’s disease”, and “Huntington’s disease” were used to search four disease-gene resources. In the GeneCards database (https://www.genecards.org/), genes with a relevance score greater than 5 were selected. From the Online Mendelian Inheritance in Man (OMIM) database (https://omim.org/), only genes with a confirmed Entrez Gene ID were retained. In the Comparative Toxicogenomics Database (CTD) (https://ctdbase.org/), genes labeled with direct evidence tags such as “marker” or “mechanism” were extracted. For the Therapeutic Target Database (TTD) database (https://db.idrblab.net/ttd/), disease-specific keywords were employed to retrieve relevant gene sets. The gene sets obtained from these databases were then visualized using a Venn diagram generated by Venny 2.1.0 (https://bioinfogp.cnb.csic.es/tools/venny/) to illustrate their overlaps. Finally, the union of all gene sets across the four databases was used to capture potential disease-related targets comprehensively.

### Construction of the protein–protein interaction network

The intersection of the predicted compound targets and the disease-associated genes was determined to obtain a set of overlapping genes. This gene set was then submitted to the STRING database [32] (https://string-db.org/) with Homo sapiens specified as the organism and a confidence score threshold of ≥ 0.4 to construct a protein–protein interaction (PPI) network. The resulting interaction data were exported in TSV format and subsequently visualized using the “*Network Analyzer*” tool in Cytoscape 3.10.3 to generate the target interaction map.

### Functional enrichment analysis of overlapping targets

To further investigate the biological functions of the overlapping drug-disease targets, Gene Ontology (GO) enrichment analysis and Kyoto Encyclopedia of Genes and Genomes (KEGG) pathway enrichment analysis were performed using the “*clusterProfiler*” package in R. Enrichment P values were adjusted for multiple comparisons using the Benjamini–Hochberg method, and terms with adjusted P < 0.05 were considered significant. GO and KEGG enrichment results were visualized using bar plots. The top 10 terms within each GO ontology—biological process (BP), cellular component (CC), and molecular function (MF)—and the top 20 KEGG pathways were selected according to their adjusted P values.

### Construction of the “drug-compound–target–pathway–disease” network

To elucidate the relationships among active compounds, target proteins, and disease pathway**s, a “drug–compound–target–pathway–disease” net**work was constructed. The identified active constituents of *C. asiatica*, the overlapping drug–disease target genes, and the top 10 enriched KEGG pathways were imported into Cytoscape 3.10.3 to visualize the network. Distinct colors distinguished different node types to represent the various components within the network clearly.

### Transcriptomic datasets acquisition and differentially expressed genes (DEGs) analysis

To explore disease-specific gene expression patterns, we obtained transcriptomic profiles for PD, AD, and HD from the Gene Expression Omnibus (GEO) database. For PD, three datasets—GSE20163 [33], GSE20164 [33] (both Affymetrix Human Genome U133A Array), and GSE26927 [34] (Illumina humanRef-8 v2.0 BeadChip)—were merged to be used as the discovery dataset, encompassing substantia nigra samples from PD patients (n = 8, 6, and 8, respectively) and NCs (n = 9, 5, and 12, respectively). The dataset GSE7621 [35] (Affymetrix Human Genome U133 Plus 2.0 Array; 9 PD and 16 NC samples) served for the independent validation. For AD, two microarray datasets, GSE5281 [36] (Affymetrix Human Genome U133 Plus 2.0 Array) and GSE36980 [37] (Affymetrix Human Gene 1.0 ST Array), comprising hippocampal tissue from AD patients (n = 13 and n = 10, respectively) and matched normal controls (NCs; n = 10 each, respectively), were merged to be used as the discovery dataset. The dataset GSE118553 [38] (GPL10558 Illumina HumanHT-12 V4.0 expression beadchip; 37 AD and 24 NC samples) was used for the independent validation dataset. Raw microarray data were processed in RStudio (version 2025.05.0 + 496) using quantile normalization via the *normalizeBetweenArrays()* function in the *limma* package, and batch effects were mitigated with the *ComBat* algorithm from the sva package. Principal component analysis (PCA) was performed to assess dataset-related separation before and after batch correction. Differentially expressed DEGs were identified using *limma* with thresholds of |log2(Fold Change)| > 0.5 and p-value < 0.05. For HD, the RNA-seq dataset GSE64810 [39] (Illumina HiSeq 2000; 20 HD and 49 NC samples) was analyzed. GSE64810 was used as the discovery dataset, whereas HD and control samples from GSE33000 [40] (GPL4372 Rosetta/Merck Human 44k 1.1 microarray; 157 HD and 157 NC samples) were used as the independent validation dataset. For the Next-Generation Sequencing data, differential expression analysis was conducted using the *DESeq2* package on the Galaxy web platform (https://usegalaxy.org/), following the methods described in our previous work [41]. Genes were identified as DEGs with criteria of |log2(Fold Change) | > 0.5 and p-value < 0.05. Benjamini–Hochberg FDR-adjusted P values were reported in the supplementary DEG tables.

### Machine learning-based feature selection for hub target genes

To identify the hub genes, an intersection was taken among the predicted compound targets, disease-associated genes, and DEGs. The overlapping gene set was further refined using multiple machine learning algorithms, including least absolute shrinkage and selection operator (LASSO) regression, support vector machine–recursive feature elimination (SVM-RFE), and random forest (RF) analysis. Candidate genes identified by all three algorithms were defined as machine-learning-prioritized hub genes potentially associated with *C. asiatica*-related disease networks. SVM-RFE was implemented in RStudio (version 2025.05.0 + 496) using the *e1071* package, with five-fold cross-validation used to estimate error and accuracy for each feature subset size; the minimum error rate determined the optimal number of features, and the corresponding genes were retained. LASSO regression was performed using the glmnet package in RStudio, with five-fold cross-validation to select the optimal lambda value, and genes with non-zero coefficients were extracted. RF analysis was conducted using the *randomForest* package in RStudio; importance scores were averaged over 1,000 iterations, and genes with scores greater than 1.0 were selected as candidates. Finally, a Venn diagram was generated using the *VennDiagram* package in RStudio to illustrate the overlap among the three feature selection approaches, and the consensus genes were defined as the final hub gene set. Candidate genes consistently identified by LASSO, SVM-RFE, and RF were defined as machine-learning-prioritized hub genes. Their reproducibility and discriminatory performance were subsequently assessed by expression validation and receiver operating characteristic (ROC) analysis in independent validation datasets.

### External validation and ROC analysis of hub genes

The robustness of the machine-learning-prioritized hub genes was further evaluated using ROC analysis. Single-gene ROC curves were first generated in the discovery dataset to assess the apparent discriminatory performance of each hub gene, and the analysis was then repeated in the independent validation dataset. To evaluate the combined performance of the hub gene set, a multigene logistic regression model was constructed using the final hub genes. Internal robustness was assessed by five-fold cross-validation in the discovery dataset. For external validation, the multigene model was trained in the discovery dataset and applied to the independent validation dataset to generate predicted probabilities for each sample. ROC curves, area under the curve (AUC) values, and 95% confidence intervals were calculated using the *pROC* package in R.

### Molecular docking analysis

Molecular docking was performed to assess predicted ligand–target interactions between the selected *C. asiatica* constituents and disease-specific hub targets. Protein structures were retrieved from the RCSB Protein Data Bank (PDB; https://www.rcsb.org/), and ligand structures were retrieved from PubChem (https://pubchem.ncbi.nlm.nih.gov/). Docking was performed using CB-Dock2 (https://cadd.labshare.cn/cb-dock2/index.php), an automated blind-docking platform that integrates curvature-based cavity detection, AutoDock Vina-based docking, and homologous template-guided pose prediction [42]. For each receptor–ligand pair, the server automatically defined candidate cavities and docking-box coordinates and ranked the resulting poses by Vina score. The cavity with the most negative score was selected for visualization; where cavities had identical best scores, the lowest cavity was retained, and all co-best solutions were reported. Each receptor–ligand pair was resubmitted three times using identical input files. Cavity ranking, docking-box parameters, Vina scores, and contact-residue profiles were identical across submissions; therefore, one representative output was retained without statistical averaging. Representative docking results and reproducibility are summarized in S1 Table in S1 File, and complete candidate-cavity outputs are provided in S2 Table in S1 File.

### Ethics statement

This study involved the secondary analysis of publicly available, de-identified transcriptomic datasets and included no new recruitment, intervention, or collection of human specimens. Ethics approval, informed-consent procedures, and tissue-bank governance reported by the investigators of the original studies were reviewed for GSE20163, GSE20164, GSE26927, GSE7621, GSE5281, GSE36980, GSE118553, GSE64810, and GSE33000. The available information, including instances in which a study-specific ethics committee, consent statement, or approval number was not reported, is summarized in S3 Table in S1 File. No additional institutional ethics approval was required for the present secondary analysis.

### Statistical analysis

All statistical analyses were performed in RStudio (version 2025.05.0 + 496), and all tests were two-sided unless otherwise specified. Genes with |log2 fold change| > 0.5 and nominal P < 0.05 were retained as exploratory differentially expressed genes for downstream integrative analyses; Benjamini–Hochberg false-discovery-rate-adjusted P values were reported in the corresponding supplementary tables. Batch effects in the merged microarray discovery datasets were adjusted using ComBat, and their removal was assessed by principal component analysis before and after correction. GO and KEGG enrichment P values were adjusted for multiple testing using the Benjamini–Hochberg method, with adjusted P < 0.05 considered significant. Each predefined hub gene was compared separately between disease and control groups using a two-sided unpaired t-test.

## Results

### Identification of active compounds and their putative targets

**Fig 1:**
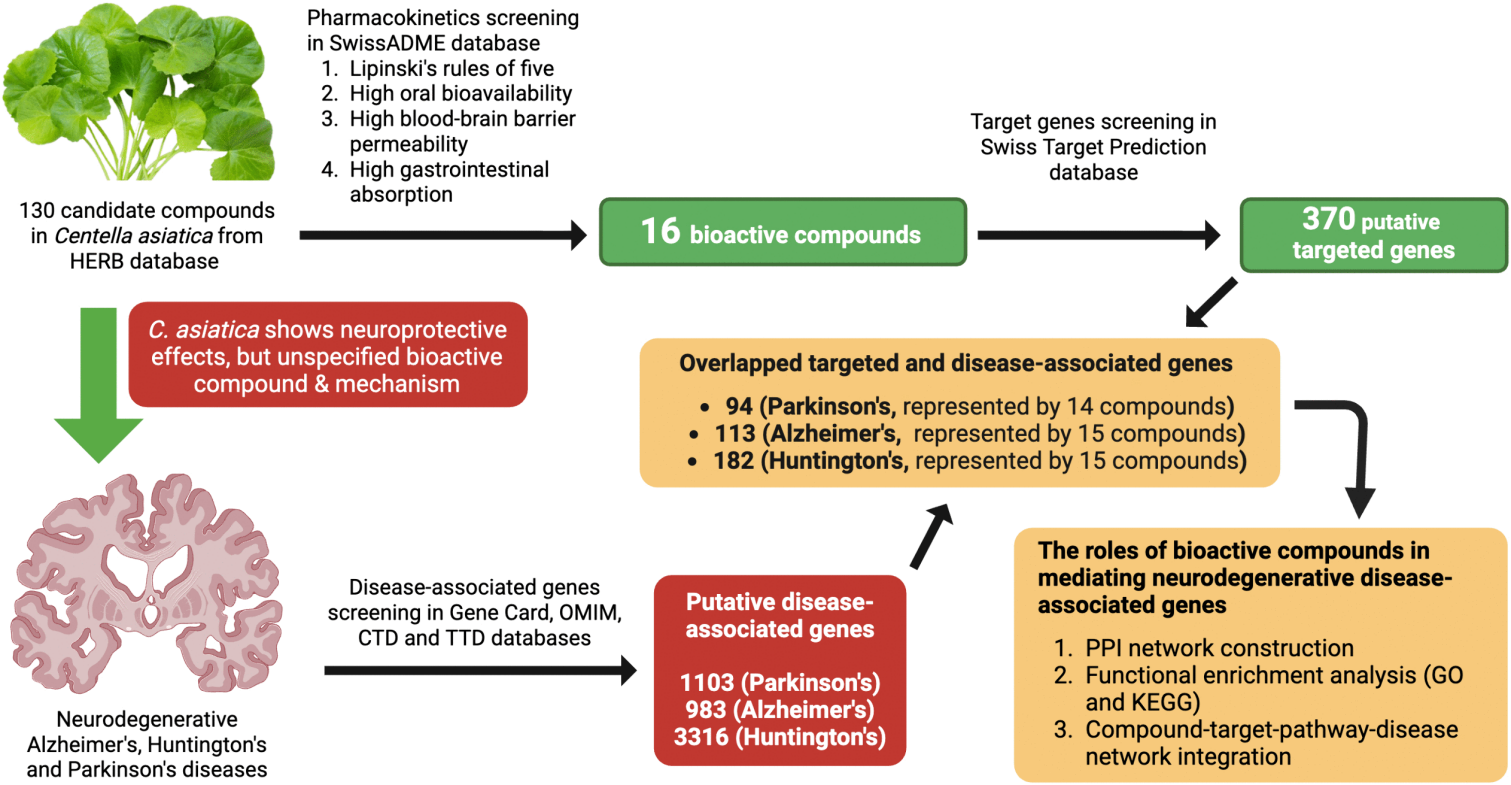
Summarized workflow of identifying C. asiatica bioactive compounds, putative C. asiatica-targeted and disease-associated genes, and downstream analyses to elucidate the roles of C. asiatica in neurodegenerative diseases. The figure was created with BioRender.

A total of 130 candidate compounds with available structural information were retrieved for *C. asiatica* from the HERB database. Based on Lipinski’s RO5, BBB permeability, and GIA parameters assessed via SwissADME, 16 candidate compounds met the screening criteria, including 1,8-Cineole (HBIN002102), (1r,2r,5s)-2,6,6-trimethylnorpinan-3-one (HBIN003059), 2-hydroxybenzoic acid (HBIN005780), 30469-22-8 (HBIN006870), 8-acetoxycentellynol (HBIN013605), anonaine (HBIN016256), Ferulic acid (HBIN026465), Glechomanolide (HBIN027930), (–)-isomenthone (HBIN030934), (l)-alpha-terpineol (HBIN032583), (r)-linalool (HBIN033265), L-menthone (HBIN033440), Menthol (HBIN034745), Pulegone (HBIN041273), Uregit (HBIN047574), and Xanthanoic acid (HBIN048422). Target prediction using SwissTargetPrediction identified a total of 370 unique drug targets associated with these 16 candidate compounds (S4 Table in S1 File) after duplicate removal. These compounds and their predicted targets were retained for downstream analyses (Fig 1).

### Multi-target mechanisms of *C. asiatica* in PD

#### Network pharmacology and enrichment analyses suggest putative multi-target mechanisms of *C. asiatica* in PD.

**Fig 2:**
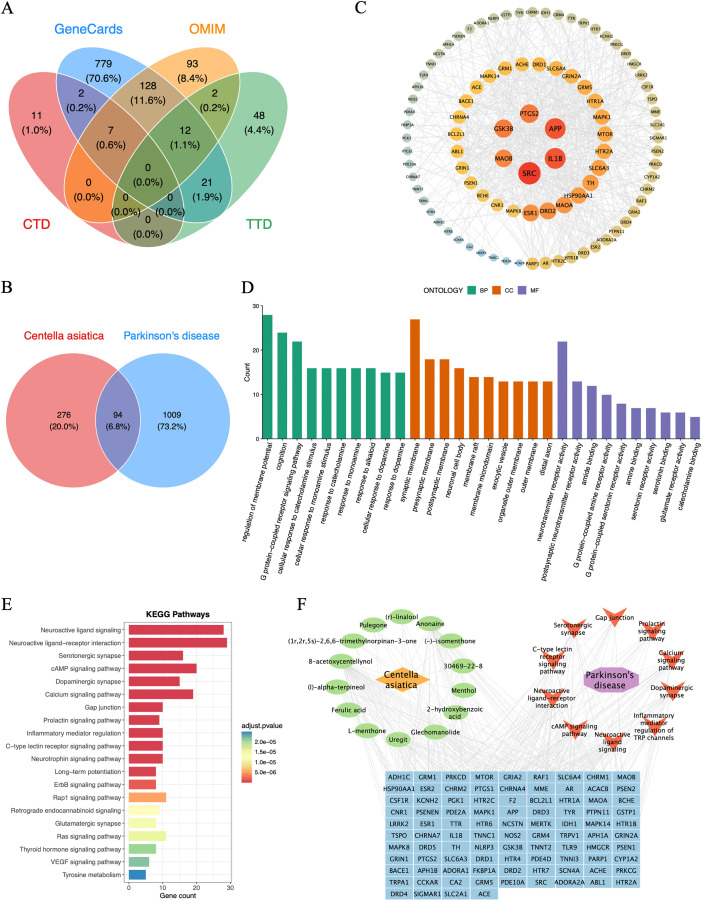
Identification, Functional Enrichment, and Network Construction of *C. asiatica*–Associated Targets in PD. (A) Venn diagram of PD-related genes retrieved from four databases (GeneCards, OMIM, TTD, and CTD). (B) Overlap between predicted *C. asiatica* targets and PD-associated genes. (C) Visualization of the overlapping genes PPI network. (D) GO enrichment analysis (BP, MF, and CC) for the overlapping genes. (E) KEGG enrichment analysis of the overlapping genes. (F) Compound–target–pathway–disease network linking 14 of the 16 screened *C. asiatica* constituents to 94 overlapping compound–PD targets and the top 10 enriched PD-related KEGG pathways.

Integration of PD-related targets from the GeneCards, OMIM, TTD, and CTD databases identified 1,103 unique genes implicated in PD pathology (Fig 2A). Venn diagram analysis revealed 94 shared targets between the predicted targets of *C. asiatica* and the PD-associated gene set (Fig 2B), suggesting that these overlapping nodes may represent candidate molecular links between *C. asiatica* constituents and PD-related processes.

To further dissect the interactions among these common targets, a PPI network was constructed using the STRING database with a confidence threshold of ≥ 0.4 and visualized in Cytoscape 3.10.3 (Fig 2C). The resulting PPI network comprised 94 nodes interconnected by 716 edges, highlighting the dense interaction landscape underlying the potential therapeutic network of *C. asiatica*. In this graph, each node represents an individual protein, whereas edges indicate physical or functional interactions. Node size and color intensity correlate positively with degree centrality, reflecting each protein’s topological importance. Notably, the ten nodes with the highest degree centrality by degree were identified as SRC, IL1B, APP, MAOB, PTGS2, GSK3B, MAOA, ESR1, DRD2, and HSP90AA1, suggesting that these proteins may serve as candidate regulatory nodes in C. asiatica-associated PD networks.

To gain insights into the functional relevance of these targets, GO and KEGG pathway enrichment analyses were carried out. The most significantly enriched GO terms are shown in Fig 2D. GO terms under the BP category predominantly involved regulation of membrane potential, cognition, G protein-coupled receptor signaling pathway, response to monoamine, cellular response to dopamine, and response to dopamine. In CC, enriched terms were mainly associated with the synaptic membrane, presynaptic membrane, postsynaptic membrane, and exocytic vesicle. For MF, the targets were enriched for neurotransmitter receptor activity, postsynaptic neurotransmitter receptor activity, G protein-coupled serotonin receptor activity, G protein-coupled amine receptor activity, and amine binding. KEGG pathway enrichment (Fig 2E) ranked the top 20 pathways based on adjusted P value, with prominent pathways including Neuroactive ligand signaling pathway, Neuroactive ligand–receptor interaction, Serotonergic synapse, cAMP signaling pathway, Dopaminergic synapse, and Calcium signaling pathway.

To comprehensively illustrate the relationships among the candidate compounds, overlapping targets, enriched pathways and PD, an integrated “drug–compound–target–pathway–disease” network was constructed using Cytoscape (Fig 2F). Of the 16 screened *C. asiatica* compounds, 14 were linked to at least one of the 94 targets shared between the predicted compound targets and PD-associated genes and were therefore included in the network. The remaining two compounds had no predicted targets within the PD-overlapping target set and were not represented. The final network comprised 14 compounds, 94 overlapping targets, and the top 10 PD-related KEGG pathways, illustrating the predicted multi-compound, multi-target, and multi-pathway architecture of *C. asiatica* in PD.

#### Machine-learning-based prioritization of candidate hub targets of *C. asiatica* in PD.

**Fig 3:**
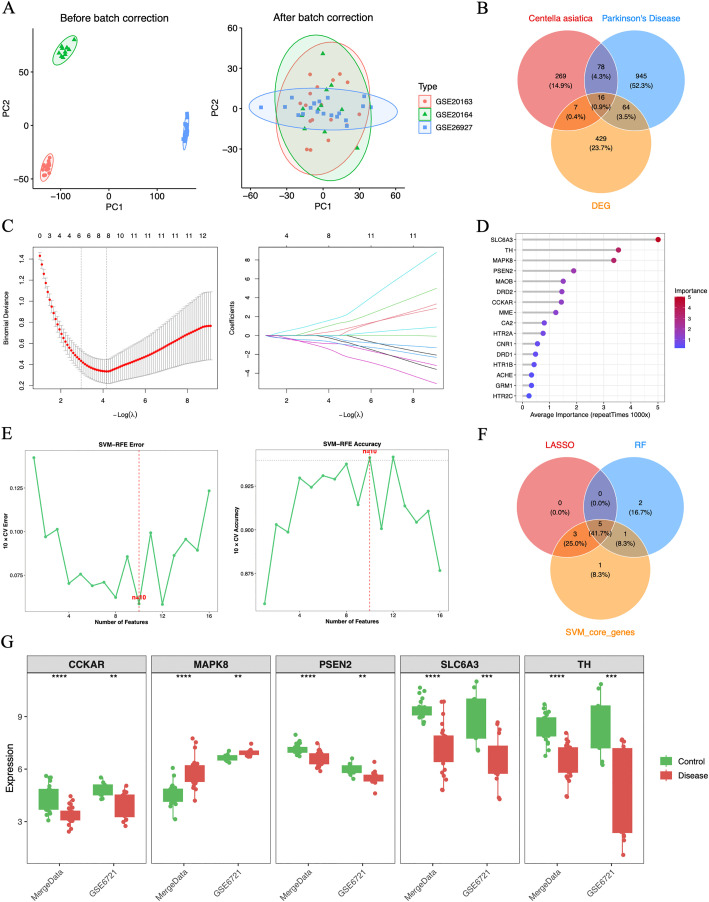
Identification and validation of hub genes associated with *C. asiatica* targets in PD. (A) Visualization of sample clustering before and after batch correction of different datasets using PCA analysis. (B) Venn analysis for the overlapping genes among *C. asiatica* targets in PD, PD-related genes, and DEGs. (C) LASSO regression analysis of hub genes in overlapping genes. (D) RF analysis of hub genes in overlapping genes. (E) SVM-RFE analysis of hub genes in overlapping genes. (F) Venn diagram of overlapping genes among LASSO regression, RF, and SVM analyses. (G) Gene expression analysis of hub genes in the merged discovery dataset and the independent validation dataset GSE7621.

To pinpoint the candidate hub targets for *C. asiatica* in Parkinson’s disease, microarray expression profiles from the substantia nigra of PD patients (datasets GSE20163, GSE20164, and GSE26927) were integrated as the discovery dataset for differential expression analysis. Principal component analysis showed that samples clustered primarily by dataset before batch correction. In contrast, dataset-driven separation was reduced after ComBat correction, supporting effective batch-effect mitigation in the merged PD discovery dataset (Fig 3A). In the combined PD discovery dataset, 516 differentially expressed genes were identified, including 197 upregulated and 319 downregulated genes. Subsequent Venn diagram analysis of overlapping *C. asiatica* targets, PD-associated genes, and DEGs revealed 16 overlapping genes (Fig 3B).

To refine these candidate genes, three machine learning algorithms were employed for feature selection. LASSO regression identified eight potential hub genes: PSEN2, MAPK8, SLC6A3, HTR2A, HTR1B, DRD1, TH, and CCKAR (Fig 3C). The RF model ranked feature importance and retained eight genes with a mean importance score exceeding 1.0: SLC6A3, TH, MAPK8, PSEN2, MAOB, DRD2, CCKAR, and MME (Fig 3D). SVM-RFE yielded ten hub genes: MAPK8, CCKAR, SLC6A3, DRD1, GRM1, MAOB, PSEN2, TH, HTR1B, and HTR2A (Fig 3E). The intersection of the three feature sets across these three methods consistently prioritized five hub genes—PSEN2, MAPK8, SLC6A3, TH, and CCKAR (Fig 3F). ROC analysis further supported the discriminatory performance of the machine-learning-prioritized PD hub genes. Single-gene discriminatory performance was assessed in both the merged discovery dataset and GSE7621. The multigene model was evaluated by five-fold cross-validation in the discovery dataset and was subsequently applied to GSE7621 for external validation (S1 Fig in S1 File). The externally validated multigene model achieved an AUC of 0.965 (95% CI, 0.892–1.000) (S5 Table in S1 File). Expression profiling showed that MAPK8 was upregulated, whereas CCKAR, PSEN2, SLC6A3, and TH were downregulated in both the merged discovery dataset and the independent GSE7621 validation dataset (Fig 3G).

#### Molecular docking suggests favourable predicted interactions between *C. asiatica* compounds and PD-related hub targets.

**Fig 4:**
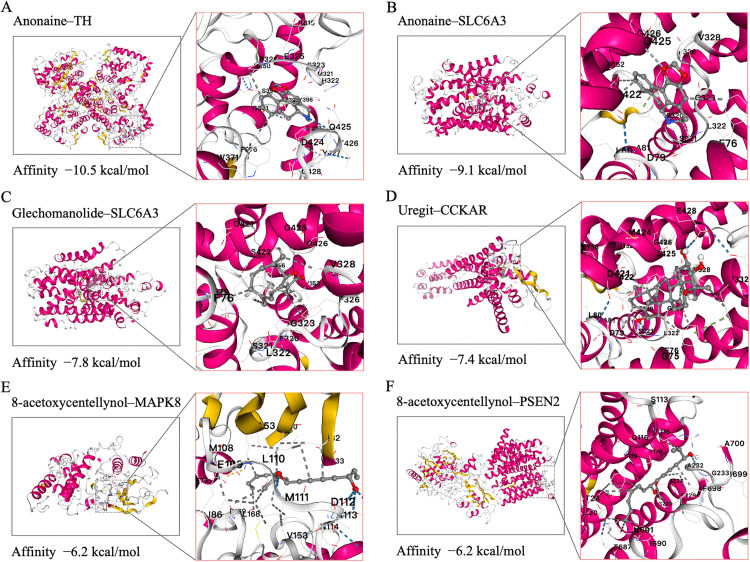
Molecular docking of four candidate *C. asiatica* compounds across six predicted PD-related ligand–target pairs. (A-F) Molecular docking results with the lowest predicted Vina docking score were selected for visualization: anonaine–TH, anonaine–SLC6A3, Glechomanolide–SLC6A3, Uregit–CCKAR, 8-acetoxycentellynol–MAPK8, and 8-acetoxycentellynol–PSEN2.

To evaluate the predicted interactions between candidate hub targets and selected compounds, molecular docking analyses were performed using five hub proteins—PSEN2 (PDB ID: 7Y5X), MAPK8 (PDB ID: 8X5M), SLC6A3 (PDB ID: 8Y2C), TH (PDB ID: 7A2G), CCKAR (PDB ID: 7F8Y)—as receptors. Four candidate compounds—anonaine, glechomanolide, uregit and 8-acetoxycentellynol—were evaluated across six predicted ligand–target pairs (Fig 4). Specifically, the predicted Vina docking scores were as follows: anonaine showed a favourable predicted Vina score to TH (−10.5 kcal/mol) and SLC6A3 (−9.1 kcal/mol). Glechomanolide interacted with SLC6A3 with a predicted Vina docking score of −7.8 kcal/mol. Uregit showed a predicted Vina docking score of −7.4 kcal/mol with CCKAR. 8-acetoxycentellynol displayed a predicted Vina docking score of −6.2 kcal/mol with MAPK8 and −6.2 kcal/mol with PSEN2.

Residue-level interaction analysis revealed key amino acid contacts that mediate these interactions. The predicted anonaine–SLC6A3 pose involved contacts with ASP79, PHE76, TYR156, PHE320, and PHE326; with TH, the interacting residues included HIS330, GLU331, GLU375, TYR370, TRP371, and PHE376. The binding of Glechomanolide to SLC6A3 involved GLY75, PHE76, VAL78, ASP79, and LEU80. For Uregit and CCKAR, predicted contact residues included CYS94, PHE97, ASN98, PRO101, and ASN102. 8-acetoxycentellynol binding to MAPK8 was mediated through GLY33, GLY38, MET108, GLU109, and VAL40; binding to PSEN2, the interacting residues included PHE682, SER683, VAL686, THR687, and TYR688. Collectively, these results support structurally plausible interactions between the selected compounds and PD-related hub targets.

### Multi-target mechanisms of *C. asiatica* in AD

#### Network pharmacology and enrichment analyses suggest putative multi-target mechanisms of *C. asiatica* in AD.

**Fig 5:**
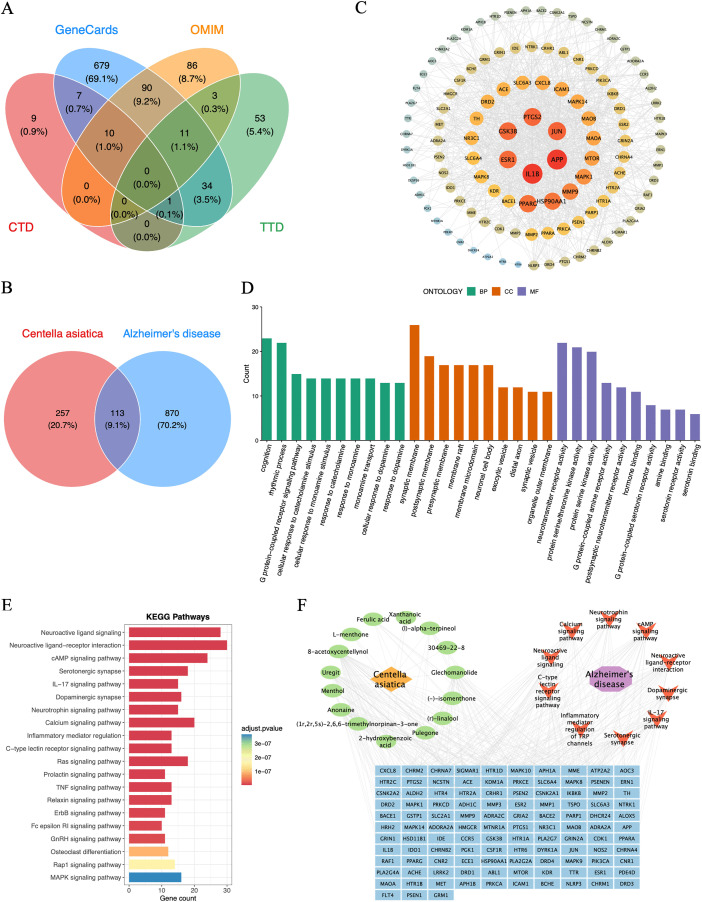
Identification, Functional Enrichment, and Network Construction of *C. asiatica*–Associated Targets in AD. (A) Venn diagram of AD-related genes retrieved from four databases (GeneCards, OMIM, TTD, and CTD). (B) Venn analysis for the overlapping genes of *C. asiatica* targets in AD and AD-related genes. (C) Visualization of the overlapping genes PPI network. (D) GO enrichment analysis (BP, MF, and CC) for the overlapping genes. (E) KEGG enrichment analysis of the overlapping genes. (F) The “drug–compound–target–pathway–disease” network linking 15 of the 16 screened C. asiatica constituents to 113 overlapping compound–AD targets and the top 10 enriched AD-related KEGG pathways.

A total of 983 unique AD-related target genes were identified by integrating data from the GeneCards, OMIM, TTD, and CTD databases (Fig 5A). Intersection analysis using a Venn diagram revealed that 113 targets overlapped between the predicted targets of *C. asiatica* and AD-associated genes (Fig 5B), indicating candidate molecular nodes potentially linking *C. asiatica* constituents to AD-related pathological processes.

To further characterize these 113 overlapping targets, PPI analysis was performed using the STRING database with a confidence score threshold of ≥ 0.4, and the resulting network was visualized in Cytoscape 3.10.3 (Fig 5C). The final PPI network comprised 113 nodes and 1,071 edges, highlighting an extensive interaction landscape among these targets. In the network, each node represents a protein, and edges indicate direct or indirect interactions. Node size and color saturation correlate positively with degree centrality. Notably, the top 10 hub proteins with the highest degree values were IL1B, APP, JUN, PTGS2, GSK3B, ESR1, PPARG, HSP90AA1, MMP9, and MAPK1, suggesting they may be candidate regulatory nodes in *C. asiatica*-associated AD networks.

To elucidate the biological functions associated with these intersecting targets, GO and KEGG pathway enrichment analyses were performed. The top 10 enriched terms within each GO ontology are shown in Fig 5D. GO analysis indicated that, within the BP category, the targets were predominantly involved in G protein-coupled receptor signaling pathway, response to catecholamine, response to monoamine, cognition, and response to dopamine. In the CC category, they were primarily associated with the synaptic membrane, presynaptic membrane, postsynaptic membrane, neuronal cell body, exocytic vesicle, and synaptic vesicle. Regarding MF, the targets were enriched in neurotransmitter receptor activity, G protein-coupled amine receptor activity, postsynaptic neurotransmitter receptor activity, and protein serine/threonine kinase activity. KEGG pathway enrichment (Fig 5E) highlighted the top 20 pathways ranked by adjusted P value, with key pathways including Neuroactive ligand signaling pathway, Neuroactive ligand–receptor interaction, cAMP signaling pathway, Serotonergic synapse, Calcium signaling pathway, IL-17 signaling pathway, Neurotrophin signaling pathway, and Inflammatory mediator regulation.

Furthermore, a drug–compound–target–pathway–disease network was subsequently assembled in Cytoscape to integrate the predicted molecular relationships relevant to AD (Fig 5F). Among the 16 screened *C. asiatica* constituents, 15 were connected to at least one of the 113 targets shared between the compound-target and AD-associated gene sets and were therefore retained in the network. One constituent was not represented because none of its predicted targets occurred within the AD-overlapping target set. The resulting network linked 15 compounds, 113 overlapping targets, and the top 10 enriched AD-related KEGG pathways, illustrating a predicted multi-compound, multi-target, and multi-pathway network involving multiple compounds, molecular targets, and biological pathways.

#### Machine-learning-based prioritization of candidate hub targets of *C. asiatica* in AD.

**Fig 6:**
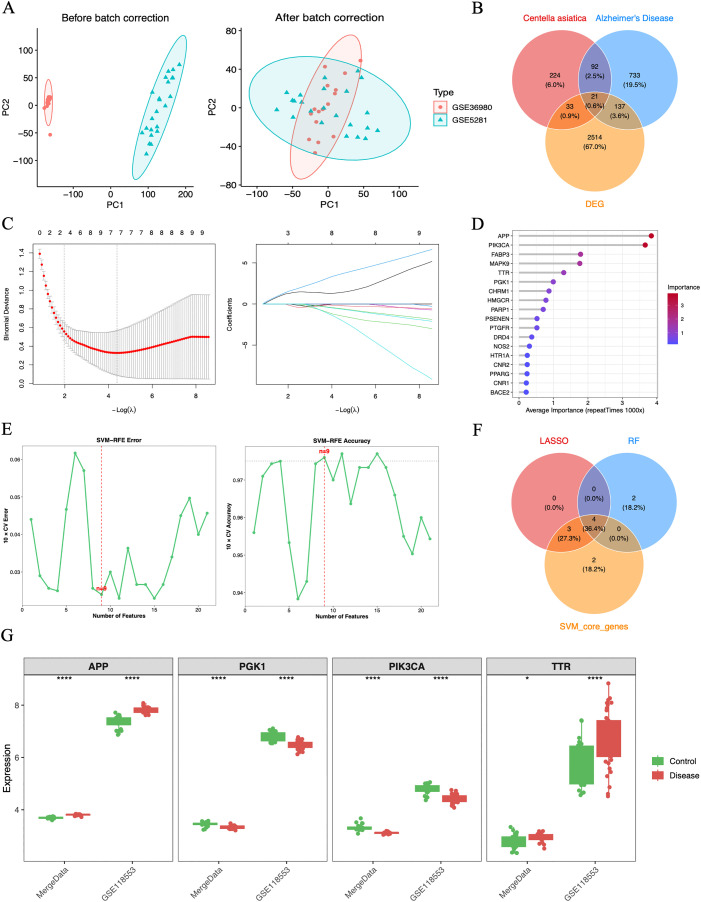
Identification and validation of hub genes associated with *C. asiatica* targets in AD. (A) Visualization of sample clustering before and after batch correction of different datasets using PCA analysis. (B) Venn analysis for the overlapping genes among *C. asiatica* targets in AD, AD-related genes, and DEGs. (C) LASSO regression analysis of hub genes in overlapping genes. (D) RF analysis of hub genes in overlapping genes. (E) SVM-RFE analysis of hub genes in overlapping genes. (F) Venn diagram of overlapping genes between LASSO regression, RF, and SVM. (G) Gene expression levels of hub genes were performed in the discovery dataset and validation dataset GSE118553.

To prioritize candidate hub targets associated with AD, hippocampal microarray datasets GSE5281 and GSE36980 were integrated as the discovery dataset for differential expression analysis. Before batch-effect correction, principal component analysis showed that samples clustered predominantly according to dataset origin. Following ComBat adjustment, dataset-driven separation was substantially reduced, and sample distribution more closely reflected biological group status (Fig 6A). Differential expression analysis of the integrated dataset identified 2,705 genes, including 1,557 upregulated and 1,148 downregulated genes. Intersecting these DEGs with the predicted *C. asiatica* targets and AD-associated genes yielded 21 candidate genes (Fig 6B).

To refine these 21 candidates, three machine learning algorithms were applied for feature selection. LASSO regression identified seven potential hub genes: PIK3CA, PGK1, APP, HTR1A, DHCR24, TTR, and PARP1 (Fig 6C). The RF analysis ranked feature importance scores, and six genes with a score >1.0 were retained: APP, PIK3CA, FABP3, MAPK9, TTR, and PGK1 (Fig 6D). The SVM-RFE method selected nine candidate genes: APP, DHCR24, PARP1, PIK3CA, PPARG, PGK1, NOS2, CHRM1, and TTR (Fig 6E). The intersection of the feature sets generated by LASSO, RF, and SVM-RFE identified four consensus hub genes: APP, PGK1, PIK3CA, and TTR (Fig 6F). The robustness of the machine-learning-prioritized AD hub genes was further examined by ROC analysis. Their individual discriminatory performance was evaluated in both the integrated discovery dataset and the independent dataset GSE118553. The multigene logistic model was evaluated by five-fold cross-validation in the integrated discovery dataset and subsequently tested in the independent GSE118553 dataset. The single-gene and multigene ROC curves, together with their corresponding AUC estimates, are presented in S2 Fig in S1 File. The externally validated multigene model achieved an AUC of 0.930 (95% CI, 0.865–0.995) (S5 Table in S1 File). Expression profiles of these four genes were extracted from the discovery and independent validation datasets. APP and TTR were upregulated, whereas PGK1 and PIK3CA were downregulated in both the discovery and independent validation datasets (Fig 6G).

#### Molecular docking suggests favourable predicted interactions between *C. asiatica* compounds and AD-related hub targets.

**Fig 7:**
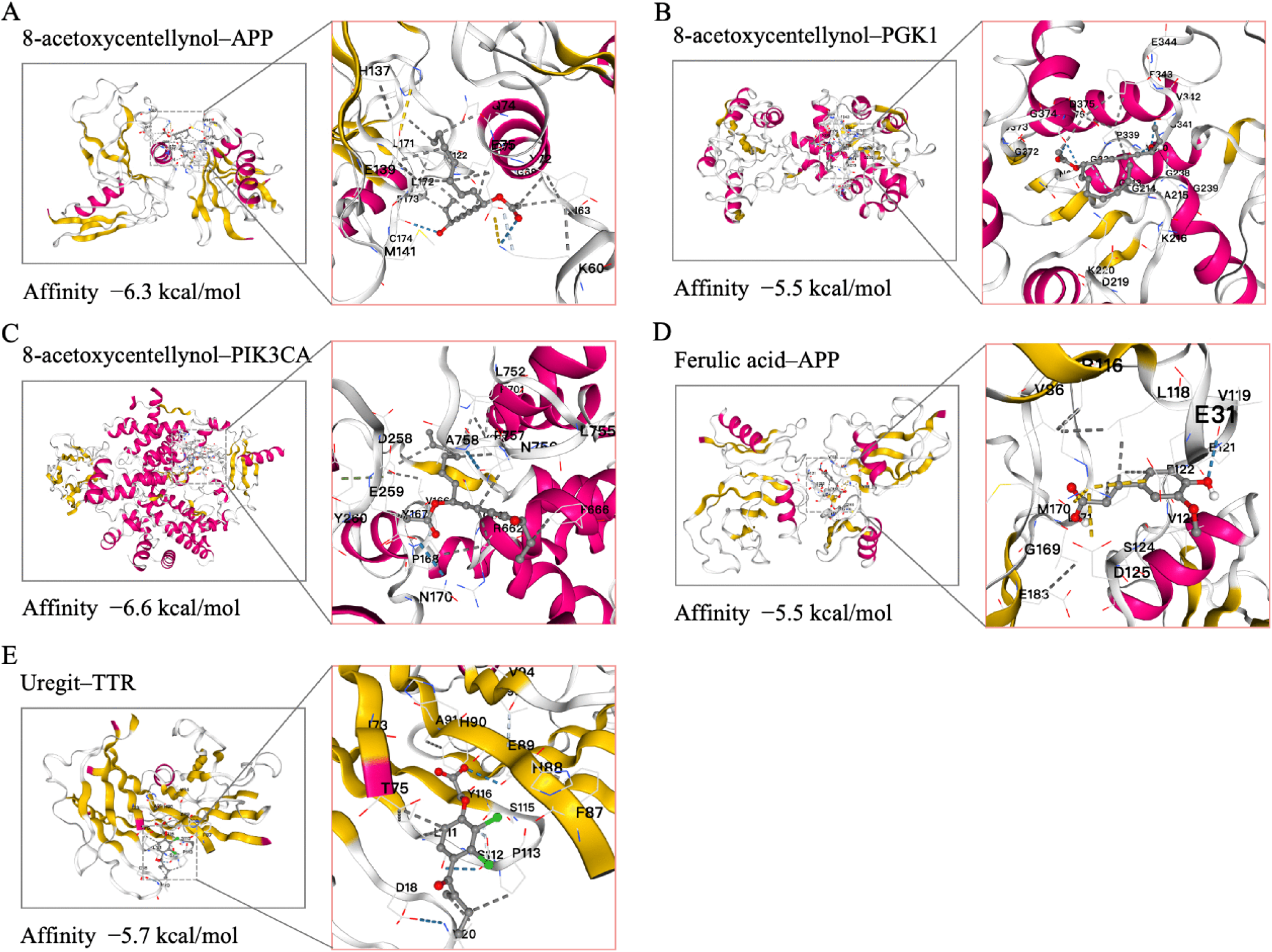
Molecular docking of three candidate *C. asiatica* compounds with four AD-related hub targets. (A–E) Representative poses for five predicted ligand–target pairs: 8-acetoxycentellynol–APP, 8-acetoxycentellynol–PIK3CA, 8-acetoxycentellynol–PGK1, ferulic acid–APP and uregit–TTR.

To evaluate predicted ligand–target interactions between key targets and active compounds, molecular docking was performed using the four hub targets—PGK1 (PDB ID: 1US8), PIK3CA (PDB ID: 7R9Y), APP (PDB ID: 4PWQ), and TTR (PDB ID: 3CBR)—as receptors. These proteins were predicted to be targets of three selected candidate constituents, namely 8-acetoxycentellynol, ferulic acid, and uregit, as ligands for CB-Dock2 analysis, using the CB-Dock2 server (Fig 7). 8-acetoxycentellynol showed predicted Vina docking scores of −6.3 kcal/mol with APP, −5.5 kcal/mol with PGK1, and −6.6 kcal/mol with PIK3CA. Ferulic acid demonstrated a favourable predicted Vina docking score of −5.5 kcal/mol with APP. Uregit displayed a predicted Vina docking score of −5.7 kcal/mol when docked with TTR.

Residue-level interaction analysis revealed that the predicted 8-acetoxycentellynol–APP pose involved contacts with residues THR83, ASN84, GLY120, GLU121, and PHE122; with PGK1, key interacting residues were GLY213, ASP219, LYS216, LYS220, and LEU223; and with PIK3CA, interactions involved TRP424, PRO447, TYR836, GLU849, and ARG818. Ferulic acid interacted with the APP transmembrane domain through critical residues ALA30, GLU31, PRO32, VAL47, and LEU80. The predicted uregit–TTR pose involved contacts with LEU17, VAL20, SER23, THR75, and ALA91. These docking results indicate structurally plausible interactions between the selected compounds and AD-related hub targets; however, they do not establish binding stability or biological activity and therefore require experimental validation.

### Multi-target mechanisms of *C. asiatica* in HD

#### Network pharmacology and enrichment analyses suggest putative multi-target mechanisms of *C. asiatica* in HD.

**Fig 8:**
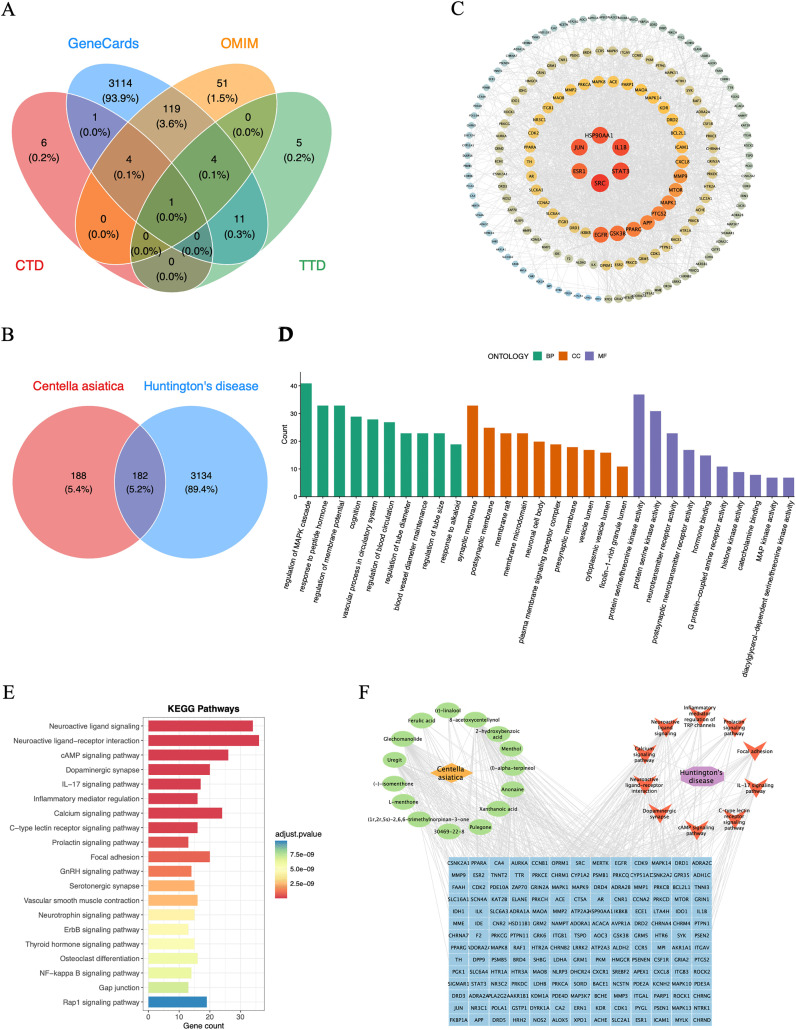
Identification, Functional Enrichment, and Network Construction of *C. asiatica*–Associated Targets in HD. (A) Venn diagram of HD-related genes retrieved from four databases (GeneCards, OMIM, TTD, and CTD). (B) Venn analysis for the overlapping genes of *C. asiatica* targets in HD and HD-related genes. (C) Visualization of the overlapping genes PPI network. (D) GO enrichment analysis (BP, MF, and CC) for the overlapping genes. (E) KEGG enrichment analysis of the overlapping genes. (F) The drug–compound–target–pathway–disease network connecting 15 of the 16 screened *C. asiatica* constituents with 182 overlapping compound–HD targets and the top 10 enriched HD-related KEGG pathways.

By integrating data from four well-established databases, GeneCards, OMIM, TTD, and CTD, we identified 3,316 unique genes associated with HD (Fig 8A). Subsequent intersection analysis using a Venn diagram revealed 182 overlapping targets between the predicted *C. asiatica* targets and HD-related genes (Fig 8B), suggesting that these shared nodes may represent candidate molecular links between *C. asiatica* constituents and HD-related pathological processes.

To elucidate the interactive landscape of these 182 common targets, we constructed a PPI network via the STRING database, applying a confidence score cutoff of≥ 0.4. Visualization and network topology analysis were performed using Cytoscape 3.10.3 (Fig 8C). The resulting PPI network comprised 181 nodes connected by 2,021 edges, highlighting a dense web of interactions. One target had no interactions in STRING under the selected confidence threshold and was therefore not represented in the final PPI network. Each node in this network corresponds to a protein, while edges represent its direct or indirect functional associations. Notably, the node size and color intensity correlate positively with degree centrality. The top ten hub proteins identified, SRC, STAT3, IL1B, HSP90AA1, JUN, ESR1, EGFR, GSK3B, PPARG, and APP**,** likely serve as high-degree nodes in the network, contributing to the predicted *C. asiatica*-associated HD network.

To gain insight into the biological roles of these intersecting targets, GO and KEGG pathway enrichment analyses were conducted. GO results (Fig 8D) demonstrated significant enrichment in BP, such as regulation of the MAPK cascade, regulation of tube diameter, response to peptide hormone, cognition, and regulation of membrane potential. Within the CC category, these targets were predominantly associated with the synaptic membrane, postsynaptic membrane, plasma membrane signaling receptor complex, serine/threonine protein kinase complex, and neuronal cell body. MF annotations highlighted enrichment in neurotransmitter receptor activity, protein serine/threonine kinase activity, hormone binding, histone kinase activity, and MAP kinase activity. KEGG pathway analysis (Fig 8E) identified the top 20 enriched pathways ranked by adjusted P value, with key signaling pathways including neuroactive ligand-receptor interaction, dopaminergic synapse, cAMP signaling pathway, IL-17 signaling pathway, Inflammatory mediator regulation, and calcium signaling pathway.

To contextualize the predicted compound–target relationships within the HD-associated pathway landscape, we constructed a comprehensive drug–compound–target–pathway–disease network using Cytoscape (Fig 8F). Fifteen of the 16 screened *C. asiatica* constituents were linked to at least one of the 182 targets shared between the predicted compound-target set and the HD-associated gene set; the remaining constituent lacked an overlapping HD-related target and was therefore not represented. The resulting network connected 15 compounds, 182 overlapping targets, and the top 10 enriched HD-related KEGG pathways, depicting a putative polypharmacological architecture in which multiple constituents converge on interconnected molecular targets and biological processes.

#### Machine-learning-based prioritization of candidate hub targets of *C. asiatica* in HD.

**Fig 9:**
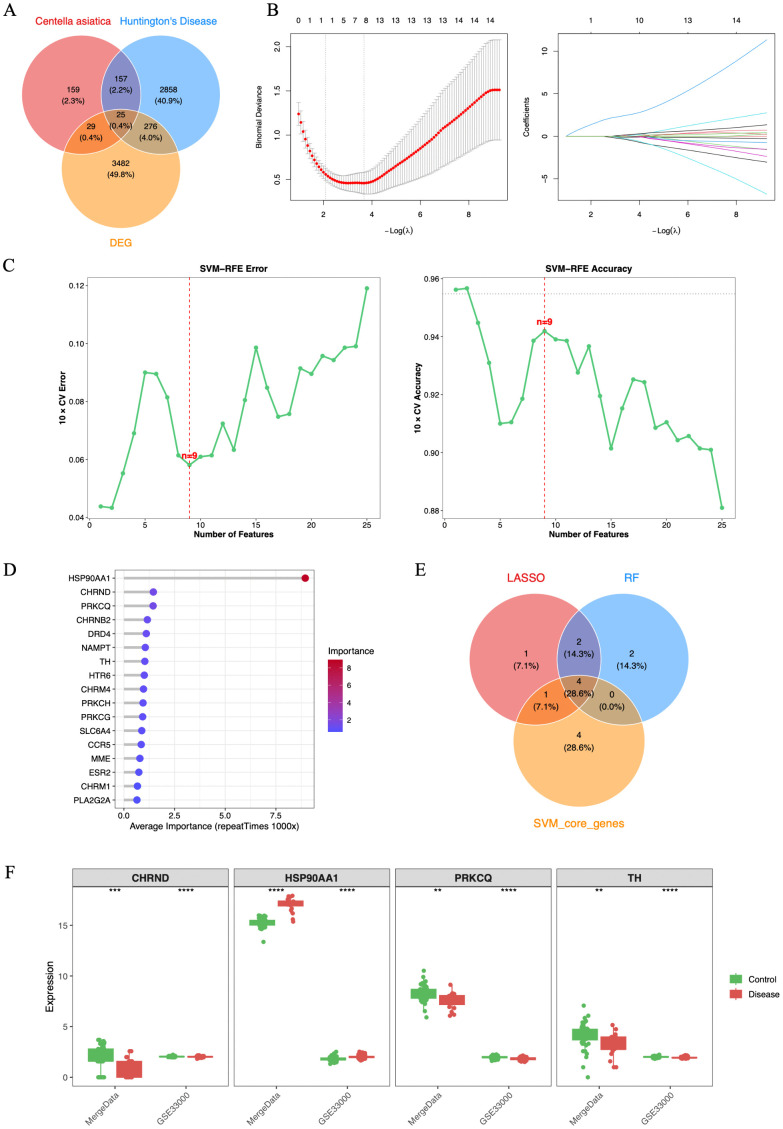
Identification and validation of hub genes associated with *C. asiatica* targets in HD. (A) Venn analysis for the overlapping genes among *C. asiatica* targets in HD, HD-related genes, and DEGs. (B) LASSO regression analysis of hub genes in overlapping genes. (C) SVM-RFE analysis of hub genes in overlapping genes. (D) RF analysis of hub genes in overlapping genes. (E) Venn diagram of overlapping genes among LASSO regression, RF, and SVM analyses. (F) Gene expression analysis of hub genes in the discovery dataset GSE64810 and the independent validation dataset GSE33000.

To identify candidate hub targets of ***C. asiatica*** in HD, we performed differential expression analysis on the next-generation sequencing dataset GSE64810, derived from the cerebral cortex of HD patients. This analysis yielded 3,811 DEGs, including 1,813 upregulated and 1,998 downregulated genes. Overlapping analysis integrating *C. asiatica* targets, HD-associated genes, and DEGs identified 29 common genes (Fig 9A).

Subsequently, three machine learning approaches were employed to prioritize these candidates. The LASSO regression model identified eight hub genes, including HTR6, NAMPT, ADRA2B, TH, CHRND, HSP90AA1, PRKCQ, and CCR5 (Fig 9B). The SVM-RFE selected nine important genes: HSP90AA1, ADRA2B, CHRND, PRKCQ, ESR2, TH, PRKCH, CHRM1, and PLA2G2A (Fig 9C). The RF analysis ranked gene importance and retained eight genes with mean importance scores above 1.0, comprising HSP90AA1, CHRND, PRKCQ, CHRNB2, DRD4, NAMPT, HTR6, and TH (Fig 9D). The intersection of the feature sets generated by LASSO, SVM-RFE, and RF identified four consensus hub genes—TH, CHRND, HSP90AA1, and PRKCQ —prioritized as candidate hub targets (Fig 9E). ROC-based evaluation was subsequently used to assess the reproducibility of the machine-learning-prioritized HD hub genes. The discriminatory capacity of each gene was examined in the GSE64810 discovery dataset and independently assessed in GSE33000. For the combined gene set, internal performance was estimated by five-fold cross-validation in GSE64810, after which the discovery-trained logistic regression model was applied to GSE33000 for external testing. The single-gene and multigene ROC curves, together with their corresponding AUC estimates, are presented in S3 Fig in S1 File. The externally validated multigene model achieved an AUC of 0.908 (95% CI, 0.874–0.941) (S5 Table in S1 File). Expression analysis showed that HSP90AA1 was upregulated, whereas CHRND, PRKCQ, and TH were downregulated in both GSE64810 and the independent GSE33000 validation dataset (Fig 9F).

#### Molecular docking suggests favorable predicted interactions between *C. asiatica* compounds and HD-related hub targets.

**Fig 10:**
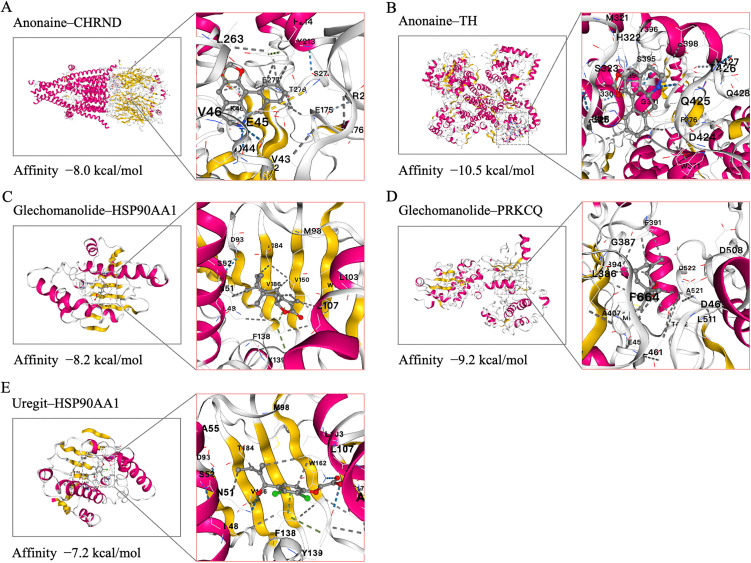
Molecular docking of three candidate *C. asiatica* compounds with four HD-related hub targets. (A–E) Representative poses for anonaine–CHRND, anonaine–TH, Glechomanolide–HSP90AA1, Glechomanolide–PRKCQ and Uregit–HSP90AA1.

To further assess the predicted interactions between the identified candidate hub targets and bioactive compounds, we conducted molecular docking analyses using four hub proteins—CHRND (PDB ID: 9DMH), HSP90AA1 (PDB ID: 5CF0), TH (PDB ID: 7A2G), and PRKCQ (PDB ID: 5F9E) —as receptors. These proteins were predicted to be targets of three candidate compounds—anonaine, glechomanolide, and uregit— as ligands for docking analysis (Fig 10). The docking simulations yielded favourable predicted docking scores, as indicated by predicted Vina docking scores: anonaine exhibited favourable predicted Vina scores with CHRND (−8.0 kcal/mol) and TH (−10.5 kcal/mol). Glechomanolide exhibited a predicted Vina docking score of −8.2 kcal/mol with HSP90AA1 and −9.2 kcal/mol with PRKCQ. Uregit showed favourable predicted Vina scores for HSP90AA1 (−7.2 kcal/mol).

Detailed residue-level analysis uncovered the predicted contact residues in these interactions. For anonaine–CHRND, key contacts were mapped to residues GLN39–VAL46, TRP176, TYR213, and PHE214. It’s predicted to bind with TH, engaging critical residues ASP190, PHE299, PHE308, TRP371, and TYR370. The docking of Uregit with HSP90AA1 highlighted contacts involving PHE22, ILE26, LEU48, ASN51, and SER52. Glechomanolide showed predicted contacts with HSP90AA1, primarily involving LEU103, ASN106, LEU107, ILE110, and GLY135. Its predicted interaction with PRKCQ involved LEU386, GLY387, PHE391, VAL394, and ALA407. These docking results provide candidate structural relationships between selected compounds and HD-related hub targets, offering hypotheses for subsequent biochemical and functional validation.

## Discussion

**Fig 11:**
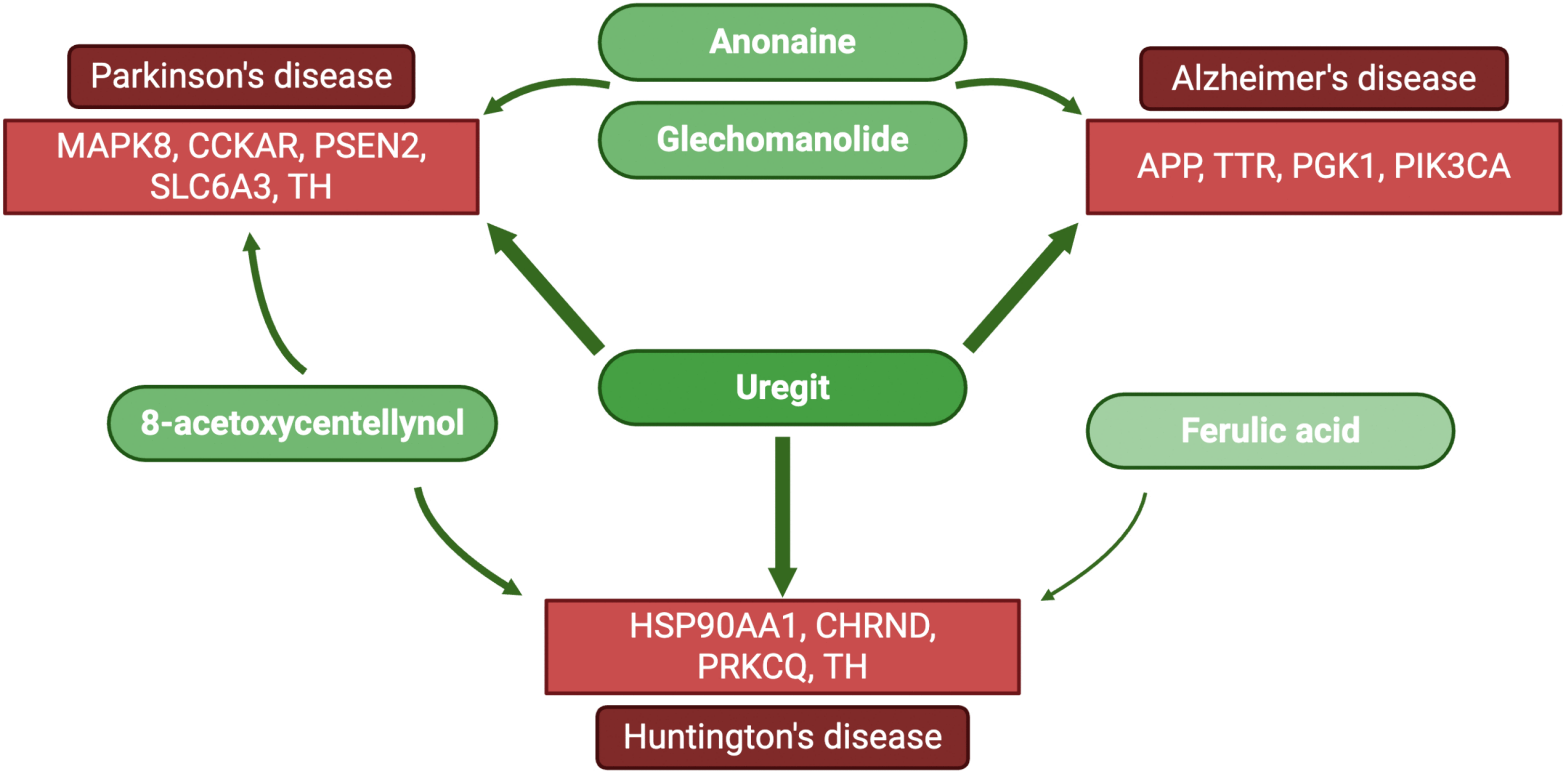
Overview of the computationally prioritized *C. asiatica* constituents, disease-specific hub targets and representative predicted ligand–target interactions in PD, AD and HD.

In this study, we integrated network pharmacology, transcriptomic analysis, machine-learning-based feature selection, independent-dataset validation, and molecular docking to prioritize candidate C. asiatica constituents and putative disease-associated targets in AD, PD, and HD. Sixteen compounds satisfied the predefined physicochemical, gastrointestinal absorption and blood–brain barrier permeability criteria, yielding 370 unique predicted targets. Integration with disease-associated genes identified 94 compound–PD targets, 113 compound–AD targets and 182 compound–HD targets. Subsequent transcriptomic and machine-learning analyses prioritized five hub genes in PD (CCKAR, MAPK8, PSEN2, SLC6A3 and TH), four in AD (APP, PGK1, PIK3CA and TTR) and four in HD (CHRND, HSP90AA1, PRKCQ and TH). Independent expression and ROC analyses provided additional support for the reproducibility of these candidate genes, whereas molecular docking identified structurally plausible interactions between selected compounds and hub targets. These findings represent hypothesis-generating computational evidence and should not be interpreted as confirmation of therapeutic efficacy or direct molecular regulation (Fig 11).

The predefined compound-selection strategy was adopted to improve methodological consistency and reduce the subjective inclusion of well-known *C. asiatica* constituents. All compounds entering the downstream analyses met the same drug-likeness, gastrointestinal absorption and blood–brain barrier permeability criteria, and no compounds failing these criteria were manually retained based on prior biological interest. Previous experimental studies have reported antioxidant, anti-inflammatory, mitochondrial-protective, and cognition-related effects of *C. asiatica* extracts and selected constituents [12,15,24–27]. However, much of the available evidence has focused on a limited number of triterpenoids, including asiatic acid, asiaticoside, madecassic acid, and madecassoside [27]. The present analysis therefore broadens the candidate space to include less extensively studied compounds such as anonaine, glechomanolide, uregit, and 8-acetoxycentellynol. Nevertheless, stringent in silico screening may exclude pharmacologically relevant constituents whose biological activity depends on metabolism, formulation, tissue exposure, or interactions within complex plant extracts.

The disease-specific drug–compound–target–pathway–disease networks further illustrated the progressive refinement of the compound set. Although 16 compounds of *C. asiatica* met the initial screening criteria, only compounds connected to at least one disease-overlapping target were represented in each network. Accordingly, 14 compounds were retained in the PD network, whereas 15 were represented in each of the AD and HD networks. This difference does not reflect inconsistent compound selection but rather the disease-specific distribution of the predicted compound targets. The resulting networks suggest that distinct subsets of C. asiatica constituents may converge on interconnected molecular targets and pathways in each neurodegenerative disease.

The disease-specific enrichment profiles were broadly consistent with established pathological features of the three disorders. In PD, enrichment in monoamine and dopamine responses, dopaminergic synapses, and membrane-potential regulation was consistent with the central involvement of nigrostriatal dopaminergic dysfunction, neuronal stress, and calcium-dependent vulnerability in disease pathogenesis [5,43,44]. In AD, the enrichment of G protein-coupled receptor-related signalling, monoaminergic responses, calcium regulation and inflammatory pathways may reflect their convergence with amyloid-associated synaptic dysfunction, neuroinflammation and calcium dyshomeostasis [45–47]. In HD, MAPK-related regulation, dopaminergic signalling and the cAMP–PKA–calcium axis are closely linked to altered striatal signalling and mutant huntingtin-associated cellular stress [6,48,49]. These enrichment results generate disease-specific pathway hypotheses for the predicted target sets but further experimental verification is needed to confirm whether *C. asiatica* directly modulates these pathways in vivo.

In PD, MAPK8 was upregulated, whereas CCKAR, PSEN2, SLC6A3, and TH were downregulated in both the discovery and independent validation datasets. MAPK8 encodes JNK1, a stress-responsive kinase implicated in oxidative injury, inflammatory signalling and apoptosis in dopaminergic neurons [50,51]. SLC6A3 encodes the dopamine transporter and regulates synaptic dopamine clearance, whereas TH catalyzes the rate-limiting step in dopamine synthesis. Reduced expression of these genes is therefore consistent with impaired nigrostriatal dopaminergic function [52,53]. CCKAR encodes the cholecystokinin A receptor, a G protein-coupled receptor involved in cholecystokinin-mediated neuropeptide signalling. Genetic studies have examined polymorphisms in the cholecystokinin system, including CCKAR, in PD cohorts, particularly in relation to neuropsychiatric manifestations such as visual hallucinations [54,55]. Although these studies do not establish CCKAR as a causal PD gene, they support the biological plausibility of the cholecystokinin receptor system as a PD-relevant neuromodulatory pathway. The reduced expression of CCKAR observed in both the discovery and validation datasets therefore warrants further investigation in disease-relevant models. Rare variants in dementia-associated genes, including PSEN2, have been reported in PD cohorts and have been associated particularly with cognitive phenotypes rather than with a confirmed causal role in PD [56]. Altered PSEN2 expression has also been shown to disrupt endolysosomal homeostasis and synaptic function in AD-relevant neuronal circuits [57]. These observations support the biological plausibility of PSEN2 as a candidate neurodegeneration-associated node, although the significance of its reduced expression in PD remains uncertain. These predicted compound–target relationships provide candidates for subsequent biochemical and functional validation.

In AD, APP and TTR were upregulated, whereas PGK1 and PIK3CA were downregulated in both the discovery and independent validation datasets. APP is directly involved in amyloid-β generation through sequential proteolytic processing, and altered APP metabolism contributes to amyloid deposition, synaptic dysfunction, and downstream neuroinflammatory responses [58]. TTR has been proposed to bind amyloid-β and restrict its aggregation and toxicity, suggesting that increased TTR expression could represent a compensatory response to amyloid-associated stress [59]. PGK1 is a glycolytic enzyme required for ATP production, and experimental activation of PGK1 has been associated with reduced protein aggregation in models of neurodegenerative disease [60]. Its reduced expression may therefore indicate impaired metabolic resilience, although this interpretation requires functional confirmation. PIK3CA encodes the p110α catalytic subunit of class I phosphoinositide 3-kinase and provides a potential link between the prioritized target set and PI3K–AKT signalling. This pathway contributes to neuronal survival, cellular metabolism, and stress responses, but the specific relevance of reduced PIK3CA expression to AD pathology remains incompletely defined [61,62]. Molecular docking suggested favourable predicted interactions between 8-acetoxycentellynol and APP, PGK1 and PIK3CA, between ferulic acid and APP and between uregit and TTR. These findings provide a structural rationale for selecting these interactions for biochemical and cellular validation.

In HD, HSP90AA1 was upregulated, whereas CHRND, PRKCQ, and TH were downregulated in both the GSE64810 discovery dataset and the independent GSE33000 cohort. HSP90AA1 encodes an inducible molecular chaperone involved in proteostasis and cellular stress responses. Modulation of the heat-shock machinery has been shown to influence mutant huntingtin aggregation and toxicity, supporting the relevance of HSP90AA1 to HD-associated protein homeostasis [63,64]. CHRND encodes the δ subunit of the nicotinic acetylcholine receptor, and altered expression of acetylcholine receptor subunits has been associated with neuromuscular-junction abnormalities and muscle dysfunction in HD models [65]. TH downregulation is consistent with previously reported disruption of dopamine synthesis and dopaminergic signalling in HD [66,67]. PRKCQ encodes protein kinase C-θ, a serine/threonine kinase involved in intracellular signal transduction and immune-cell activation. Transcriptomic profiling of patient-derived juvenile HD induced pluripotent stem cells identified PRKCQ among the downregulated transcripts associated with DNA-damage-response and apoptosis-related networks [68]. This observation is concordant with the reduced PRKCQ expression detected in the present discovery and validation datasets. Nevertheless, the cell-type-specific function of PRKCQ in HD and its contribution to mutant huntingtin-associated neurodegeneration remain poorly understood. The predicted interactions of anonaine with CHRND and TH, glechomanolide with HSP90AA1 and PRKCQ, and uregit with HSP90AA1 provide candidate structural relationships for future testing.

Several limitations should be considered. Compound and target prioritization relied on public databases and prediction algorithms, which may contain incomplete or context-dependent annotations, while bulk-tissue transcriptomic data cannot distinguish cell-intrinsic changes from differences in tissue composition. Differences in platform, sample size, and cohort characteristics may also affect the generalizability of the findings. In addition, molecular docking provides static computational predictions and does not establish biochemical affinity, target engagement, or functional activity. Future studies should therefore validate the prioritized compound–target pairs using biochemical binding assays, disease-relevant neuronal or glial models, pharmacokinetic studies and appropriate in vivo experiments.

## Conclusion

This study provides an integrative computational framework for prioritizing candidate active constituents of *C. asiatica* and their putative targets in AD, PD, and HD. By combining network pharmacology, transcriptomic analysis, machine-learning-based feature selection, and molecular docking, we prioritized candidate compounds, hub targets, and pathways potentially associated with disease-relevant molecular networks. The prioritized target networks were enriched in processes related to oxidative stress, neuroinflammation, apoptosis, synaptic signalling and cAMP- and calcium-associated pathways. These findings generate testable hypotheses for the future experimental evaluation of *C. asiatica*-derived neuroprotective candidates. Still, experimental validation in biochemical, cellular, and animal models is required to confirm their mechanistic and therapeutic relevance.

## Supporting information

S1 File S1 Fig. ROC analysis of machine-learning-prioritized hub genes and the multigene model in Parkinson’s disease. S2 Fig. ROC analysis of machine-learning-prioritized hub genes and the multigene model in Alzheimer’s disease. S3 Fig. ROC analysis of machine-learning-prioritized hub genes and the multigene model in Huntington’s disease. S1 Table. Representative CB-Dock2 docking outputs and repeat-submission concordance. S2 Table. Complete CB-Dock2 candidate-cavity outputs for all ligand–target pairs. S3 Table. Source publications, tissue provenance and original ethics and consent information for the GEO datasets. S4 Table. Candidate *C. asiatica* constituents and predicted targets. S5 Table. ROC performance of machine-learning-prioritized hub genes and multigene models.
(ZIP)
