# Supplementary material for: Integrative network pharmacology, transcriptomics, and molecular docking identify candidate Centella asiatica constituents and targets in neurodegenerative diseases
Source: PLoS One. 2026 Jul 31;21(7):e0354882. doi: 10.1371/journal.pone.0354882 (PMC13426974; doi:10.1371/journal.pone.0354882)
Supplement: S1 File — S2 Fig. ROC analysis of machine-learning-prioritized hub genes and the multigene model in Alzheimer’s disease. S3 Fig. ROC analysis of machine-learning-prioritized hub genes and the multigene model in Huntington’s disease. S1 Table. Representative CB-Dock2 docking outputs and repeat-submission concordance. S2 Table. Complete CB-Dock2 candidate-cavity outputs for all ligand–target pairs. S3 Table. Source publications, tissue provenance and original ethics and consent information for the GEO datasets. S4 Table. Candidate C. asiatica constituents and predicted targets. S5 Table. ROC performance of machine-learning-prioritized hub genes and multigene models. (ZIP) [file pone.0354882.s001.zip › S1 Fig.pdf]

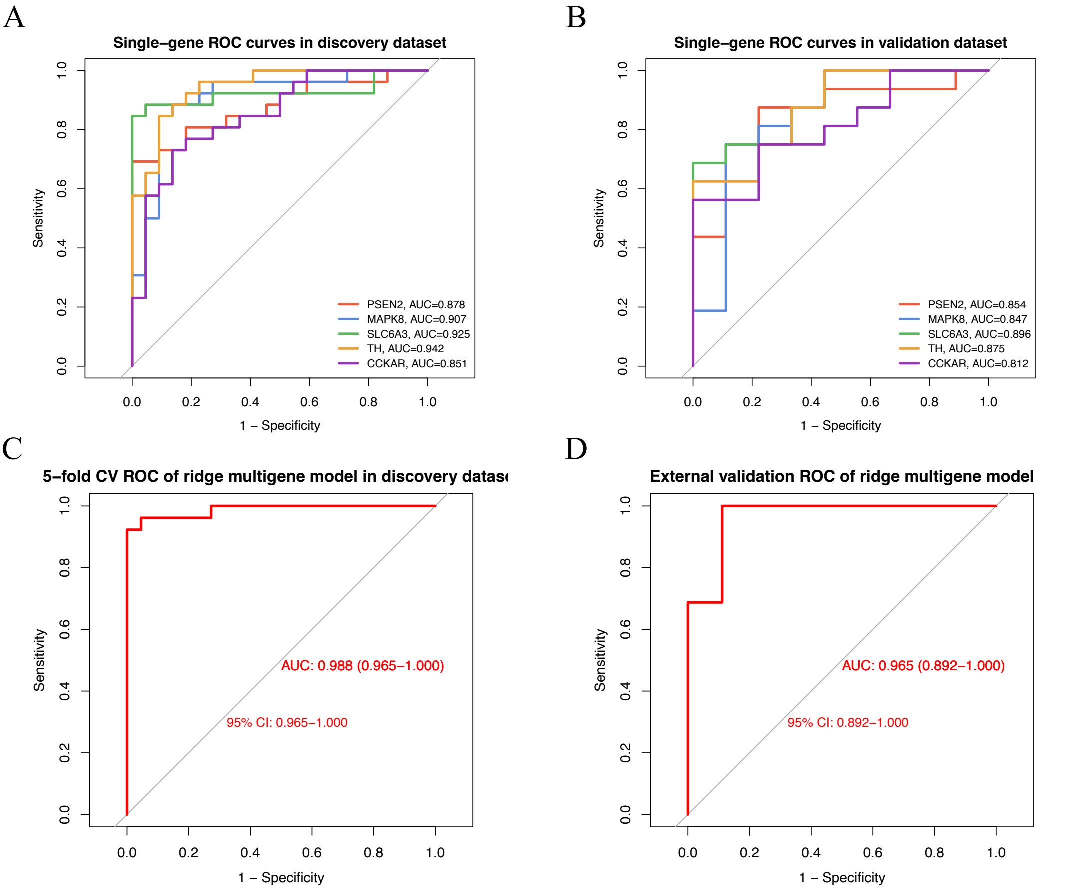

S1 Fig. ROC analysis of machine-learning-prioritized hub genes and the multigene model in Parkinson's disease. (A) Single-gene ROC curves of the five PD hub genes in the discovery dataset. (B) Single-gene ROC curves of the five PD hub genes in GSE7621. (C) Five-fold cross-validation ROC curve of the ridge multigene model in the PD discovery dataset. (D) External validation ROC curve of the ridge multigene model in GSE7621. ROC, receiver operating characteristic; AUC, area under the curve.
